# Strategies for Effective Public Health Communication in a Complex Information Environment

**DOI:** 10.1146/annurev-publhealth-071723-120721

**Published:** 2024-12-10

**Authors:** Jeff Niederdeppe, Amanda D. Boyd, Andy J. King, Rajiv N. Rimal

**Affiliations:** 1 Department of Communication, Cornell University, Ithaca, New York, USA; 2 Cornell Jeb E. Brooks School of Public Policy, Cornell University, Ithaca, New York, USA; 3 Elson S. Floyd College of Medicine, Washington State University, Spokane, Washington, USA; 4 Edward R. Murrow College of Communication, Washington State University, Pullman, Washington, USA; 5 Department of Communication and Huntsman Cancer Institute, University of Utah, Salt Lake City, Utah, USA; 6 Johns Hopkins Bloomberg School of Public Health, Johns Hopkins University, Baltimore, Maryland, USA

**Keywords:** health communication, health equity, misinformation, behavioral science, effectiveness, multilevel interventions

## Abstract

Development and amplification of effective, culturally resonant, trustworthy, and evidence-based public health communication are urgently needed. Research evidence, theory, and practical experience from within and beyond the interdisciplinary field of health communication are well-positioned to help public health authorities, researchers, and advocates navigate the complex societal challenges that influence health and well-being in global contexts. This review offers a broad overview of the field, considers what constitutes “effectiveness” versus “effects” in public health communication, and describes core concepts of public health communication as a process rather than a product. We review domains and dominant foci of public health communication research, articulate challenges for health communication to advance health and social equity and address mis-/disinformation, and offer practical guidance on message development, audience segmentation, multilevel intervention, and evaluation of communication programs. We conclude by identifying important questions for future public health communication research, interventions, and funding.

## 1. INTRODUCTION

Effective public health communication is urgently needed. Recent developments in the public communication environment have changed how people engage with information about health and health-related public policies (87, 117). The COVID-19 pandemic underscored the challenges of communicating swiftly evolving information about a novel public health threat and strategies to mitigate it (7). Scientific understanding of the scope, causes, and consequences of deteriorating health outcomes in the United States has not been met with corresponding public understanding or urgency (145). Limited public knowledge of public health principles, diminished trust in public health authorities, and declines in funding for local, state, and federal public health organizations across the globe have brought additional challenges for public health communication (34, 40, 57). The World Economic Forum lists misinformation (e.g., false information) and disinformation (e.g., false information intended to mislead) as the most severe global risk (146), and the US Surgeon General lists health misinformation among its highest-priority issues (131).

Research evidence, theory, and practical guidance from within and beyond the interdisciplinary field of health communication offer guidance in navigating these complex societal challenges. This review provides a broad overview of the field, considers what constitutes effective public health communication, and describes its core concepts. We articulate challenges for health communication to advance public health, achieve social equity, and address mis-/disinformation. We offer practical guidance on message development, audience segmentation, multilevel intervention, and evaluation of communication programs. We conclude with considerations for future public health communication research, interventions, and funding.

### 1.1. Defining Public Health Communication

Many organizations have defined health communication as a field [e.g., International Communication Association (https://www.icahdq.org/group/health); National Communication Association (https://www.natcom.org/health-communication-division)]. We find the Society for Health Communication’s definition helpful (additions in brackets): “Health communication is a multidisciplinary field of study and practice that [develops and] applies communication evidence, strategy, theory, and creativity to promote behaviors, policies, [systems], and practices that advance the health and well-being of people and populations” (121). Health communication also occurs at multiple levels, from individuals to groups, networks, organizations, media systems, and broader cultures and societies (91). Health communication involves textual, aural, oral, verbal, and/or visual communication through various channels that include interpersonal conversation (among family, friends, and others), doctor–patient interaction, social media, news, entertainment, advertising, organizational messaging, policy briefs, infographics, advocacy materials, and beyond.

We limit our focus here to *public* health communication, which is narrower than health communication writ large. By emphasizing “public,” we note the American Public Health Association’s distinction between health care and public health: “The health care industry treats people who are sick, while public health aims to prevent people from getting sick or injured in the first place. Public health also focuses on entire populations, while health care focuses on individual patients” (1). These distinctions highlight that public health is far more than communication between providers and patients and extends beyond the emphasis on health care systems that is found in the US Healthy People 2030 health communication objectives (12 of 19 of which are focused on providers or health technologies) (48).

Public health communication is also more than just communicating about health or disease. While it is often important to communicate about specific health risks of a behavior or a policy, effective public health communication may also involve messaging about health-influencing behaviors and policies without explicit focus on those health impacts. For instance, messaging about tobacco prevention among youth has at times emphasized the cost of cigarettes, social norms around smoking, or the tobacco industry’s predatory marketing to young people (35). It is also instructive to differentiate public health communication that intends to support informed decisions from communication that intends to persuade, though the two are often intertwined. Public health communication is also more than sharing research evidence with relevant audiences (112). Finally, public health communication is more than the sum of disciplinary knowledge in communication science, public health, medicine, and psychology. Rather, it integrates knowledge from many fields, which include sociology, economics, political science, public policy, data science, and computer science, as well as the recently emerging fields of health informatics, dissemination science, and infodemiology (the epidemiology of information).

### 1.2. Public Health Communication as a Process

Thinking of public health communication as a product is common (e.g., a brochure or poster designed to change people’s health behaviors). Adopting this perspective misses the dynamic nature of the communication process, which can change across contexts, over time, and between audiences. Communication that is persuasive in one context may be ineffective in another. In the climate change literature, for example, climate adaptation and mitigation behaviors are strongly associated with trust in environmental groups, institutions, and scientists (29), and level of trust itself varies across populations and countries (33). Hence, the same communication emanating from scientists may be perceived as credible by one group and dismissed by another group.

One way to conceptualize this dynamic nature of public health communication is to make allowance for factors pertaining to interactions among source, message, channel, and audience. Issues of trust and credibility are characteristics of the source that are perceived by an audience; the extent to which the information is thought to emanate from trustworthy people and institutions depends on both the source itself and the eye of the beholder. Similarly, messages can vary in what and how they communicate to whom, including their literacy burden, whether they are framed positively or negatively, and/or the extent to which they rely on facts versus stories. Some channels (e.g., brochures) allow for a significant amount of content, while others (e.g., a 30-second commercial) do not. Similarly, some (e.g., email) allow for feedback, whereas others (e.g., public health agency guidelines) do not. Finally, audience characteristics (their interests, capabilities, motivation) interact in countless ways with sources, messages, and channels. Reaching audiences with strategic messages from sources they find credible, through channels they use and trust, is neither formulaic nor static. Effectively reaching audiences requires inquiry, trial and error, and adaptations through learnings from research and evaluation.

### 1.3. What Is Effective Public Health Communication?

Public health communication interventions (e.g., campaigns) are social actions that emerge from social processes (in which various actors collaborate or compete for attention and ways of framing a topic) and occur at multiple levels (e.g., interpersonal networks, media/information systems, and broader cultural contexts) (25). Determining the effectiveness of purposeful public health communication poses a challenge for many reasons (see discussion below on evaluation), not the least of which is because many health communication studies are carried out in settings that prioritize rapid learning or internal validity over external or ecological validity. For example, some studies utilize focus groups or qualitative interviews among a (relatively) small audience to gain insights into resonant themes and messages before launching a large-scale campaign (46, 50). Other studies use randomized experiments to test or compare the effects of discrete messages (e.g., components of a strategic health communication effort), delivered in contrived settings (e.g., within a Web-based survey or in a laboratory) in shaping beliefs, attitudes, or behavioral intentions (83) or support for a policy (68). Studies in the latter camp are often characterized as testing for message effects, not effectiveness at scale or in situ. These designs are more closely aligned with what intervention research might call an efficacy trial (113). In contrast, effectiveness trials are designed to determine if an intervention (e.g., a communication campaign) works in real-world (versus ideal) conditions. Both types of research can offer insights about effective health communication but differ in how they test for effectiveness (e.g., testing an intervention component rather than a full intervention). As in many other health fields, there is also ongoing debate among health communicators about effective methods for testing the efficacy of interventions before broader dissemination and the degree to which those findings predict effectiveness in broader settings (82, 85).

Distinguishing between effectiveness and effects is helpful for having nuanced discussions and evaluations of effective public health communication (107). Broadly considered, health communication efforts having effects means that changes or differences in outcomes of interest can be attributed to those efforts. Effectiveness relates to whether stated expectations (e.g., objectives) have been achieved (106). Effectiveness is more complicated because the success of communication efforts may be judged in many ways, including absolute (e.g., outcomes are influenced as intended) or relative (e.g., communication is better or more cost-effective than other approaches at influencing outcomes) (107). Health communication efforts can be conceptualized in a two-by-two matrix of being effective or not effective and as having effects or not having effects. Identifying health communication efforts that are effective and have effects is straightforward (e.g., increased cancer screening rates in a community after introducing a program intended to produce an increase in those rates). Identifying health communication efforts that are effective but do not have effects would be exemplified by a program that reinforces existing positive behavior rates (e.g., ensuring smoking rates do not increase, mitigating increases in a population over time). Identifying efforts that are not effective but have effects can result from having effects below the level set as a goal (e.g., 44% of people completed a promoted healthy eating intervention, but the objective was to have 60% complete the intervention) or producing unintended effects (e.g., no change in healthy eating behavior but a change in community social norms) (25). Making sense of effects and effectiveness of public health communication interventions requires clear a priori specifications of the magnitude and direction of expected outcomes, a challenge given the diversity of outcomes of interest to communication researchers and practitioners.

## 2. DOMAINS OF PUBLIC HEALTH COMMUNICATION RESEARCH

### 2.1. Multilevel Outcomes and Theories of Change for Public Health Communication

Much public health communication research has focused on the extent to which efforts have influenced individual-level outcomes, including but not limited to awareness (71), behavioral intentions (55), behavior change (120), risk perceptions (83), decision-making (23), information seeking (53), message sharing (60), learning (58), trust (39), and policy support (81). Behavior change has been the most sought-after individual-level outcome, whether reducing or eliminating harmful activities (e.g., smoking) or initiating healthy activities or increasing their frequency (e.g., physical activity) (2, 64, 70, 120).

Highlighting a fear of negative health consequences is often used as a behavior change strategy (10, 105, 126), and meta-analytic evidence supports the conclusion that, on average, messages that evoke fear have a positive influence on outcomes of interest, particularly in the short term and for episodic behaviors such as screening or vaccination (126). At the same time, oft-cited theories such as the extended parallel process model (EPPM) emphasize that people are more likely to take preventive action when they both are motivated (e.g., they are aware of their risk of a particular disease) and believe they have the ability (self-efficacy) and effective means with which to act (response efficacy) (144). Some studies have found that overemphasis on risk can increase anxiety and fear to levels that are counterproductive and can cause people to ignore the communication (76, 127). Meta-analytic evidence further indicates that fear appeals have a stronger impact when they include positive response efficacy information (i.e., information about how taking action will lead to a desired positive outcome; see Reference 10).

Perceptions of risk and beliefs of efficacy are, of course, factors at the individual level. Effective public health communication, however, must also incorporate determinants at the community, organizational, and policy levels. One such factor pertains to social norms, which include beliefs about the prevalence of a behavior, also called descriptive norms, and pressure people feel to conform, called injunctive norms (28). Social norms are thought to operate at the social or cultural level (though they are often measured at the individual level and aggregated at the community level; see Reference 110 for an example of such aggregation). In the presence of high-risk social norms, for example, young adults who know about the harmful effects of their behaviors continue to engage in those behaviors, as has been observed in the domain of alcohol consumption among US college students (139). Similarly, public health communication can induce people to act through a social norms strategy that highlights others’ actions (98). However, the relationship between social norms and behaviors depends on other factors (100). For instance, the impact of social norms on behaviors is heightened in the presence of other facilitating factors, such as the perceived benefits of taking action, self-efficacy to act, presence of others in the environment, and knowledge of an appropriate course of action in a given environment (27).

There is far less public health communication scholarship on institutional or policy change as a vehicle for advancing public health. Part of this challenge stems from a limited theoretical tool kit to inform communication to support structural interventions. Many public health communication scholars advocate for health communication scholarship to be grounded in theory and, optimally, to contribute to development and refinement of theory while addressing applied health topics (37, 104). This approach regularly takes the form of using behavior change theory to identify relevant cognitive and emotional variables that predict changes in behavioral intention and behavior, and theorizing in the field has heavily centered psychological/cognitive factors over sociological/structural factors (32). Many frameworks, including the integrated model of behavioral prediction (IMBP) (36), social cognitive theory (6), the EPPM (144), and the COM-B (capability, opportunity, motivation–behavior) model (74), identify dozens of variables that predict behavior, intention, and persuasion. While several of these frameworks acknowledge that structural factors (environmental constraints in the IMBP; opportunity in the COM-B model) enable or constrain health behavior, they offer far more detail on constructs focused on predicting volitional behaviors than they offer on the mechanisms of structural impediments, and none offers guidance on how communication can influence those constraints or opportunities. At the same time, attempts to clarify the relative contributions of structural versus behavioral contributions usually conclude that health is shaped as much by social and structural factors as by individual behaviors (63, 67, 72). Ecological models also make clear that behaviors are heavily shaped by social, institutional, and structural forces (142). Yet these ecological models are also silent about pathways to changing the institutions, structures, and policies that drive health outcomes. Political science frameworks on the policy process also say little about the specific roles of communication in these processes beyond broadly acknowledging that advocacy and coalition-building are important.

One consequence of this asymmetry in theoretical development is that scholars who seek to conduct theoretically grounded research on communication for institutional or policy change have little theoretical guidance on the communication processes and outcomes that matter for policy change, despite calls for centering evidence-based policy change to address major public health concerns (16, 93). The dearth of attention to communication and policy change processes has led to a predominant focus on outcomes such as “policy support” and “policy attitudes” (81). While public opinion about policies can at times influence policy debates, there are many cases where public opinion does not translate to broad shifts in health and social policy (8, 18, 42). Changes to public policies that advance health and address health inequality may also be catalyzed by empowering and mobilizing groups and populations who stand to benefit most from these policies (9, 11). Other cases of policy change to advance health equity may occur outside of formal democratic processes through administrative changes by appointed officials (22). These officials may be influenced by media coverage, direct translation and dissemination of scientific findings, strategic advocacy via digital media, or even interpersonal communication (148).

Research that centers community mobilization and power-building as pathways to policy change in communication scholarship would call attention to the need to center the perspectives and lived experiences of populations who face the greatest health and social inequities, including communities of color, queer communities, and impoverished communities (31, 96). Increased emphasis on policy outcomes and processes would also amplify a need to consider the perspectives of policy-making actors and entities when developing research designs (e.g., pursuing samples of legislators, staffers, county executives, or other health and social administrative officials). While few scholars have explored how elected officials respond to various forms of public health communication (see, for example, 17, 95), these populations remain vastly understudied.

### 2.2. Advancing Public Health and Social Equity

Public health communication has the potential to help reduce health inequities and advance social justice. However, health communication also runs the risk of widening disparities if sources of inequality are not considered; these include access/exposure to information and systemic barriers to behavior change (134). To increase health equity through communication, practitioners and researchers can incorporate strategies that include (*a*) employing strength-based messaging and communication approaches, (*b*) incorporating and empowering historically marginalized populations in communication development and dissemination, and (*c*) considering barriers to behavior change and information access. Communication with and about historically minoritized communities has often emphasized deficits and weaknesses (133). Yet many communities have strengths that are overlooked by communicators, including strong social networks or messaging strategies grounded in historical storytelling and oral traditions. Campaigns that highlight strengths and incorporate community assets can facilitate effective communication and increase perceptions of community self-worth (49). Furthermore, when historically marginalized people are involved in communication development and dissemination, positive health outcomes often follow (3) because communities and members facing specific risks have unique insights into the barriers to health communication reception or processing.

In general, communicators need a clear understanding of the barriers to and facilitators of communication within priority populations or regions. For example, many parts of the world have poor access to Internet or cellular service; mobile health campaigns are unlikely to succeed in these areas. In some of these areas, people commonly use radio for seeking information and learning about public health (24). Without understanding the communication resources and realities of a priority population, particularly historically minoritized populations, communication campaigns may fail and potentially even increase health disparities.

Improving public health for all and advancing health equity may require different communication strategies and approaches. Some population groups have an increased risk of morbidity and mortality due to long-standing social and health inequities (142). As noted previously, strategies designed to promote overall population health without specific attention to structural barriers to change among populations that face these inequities run the risk of widening, not reducing, health disparities (31, 134). Numerous organizations around the world have called for communication practices that emphasize inclusivity and center efforts to address health inequities (45). For example, the US Centers for Disease Control and Prevention (CDC) and the World Health Organization have described considerations for communication using a health equity lens (129, 141). These principles include recognizing unique histories of racism and discrimination for minoritized populations that shape interaction with health information and systems, centering community engagement in message design, recognizing diverse and intersectional identities of people and audiences, and communicating in ways that are accessible and understandable.

### 2.3. Addressing Misinformation and Disinformation

As noted above, many governmental and nongovernmental organizations have declared mis-/disinformation as among the greatest challenges to health and social welfare (131, 146). The rampant spread of misinformation on social media during several phases of the COVID-19 pandemic (38, 114) has been well-documented. These are often purposive efforts to amplify disagreement with established scientific findings related to vaccination (15). Long-established corporate disinformation strategies by commercial marketers of unhealthy products (21, 89, 128) have also brought considerable attention to the prevalence and impact of false health information in the public communication environment (122, 124). Many people hold inaccurate beliefs about a wide range of health-related topics (69), and many studies link misinformation to negative health-related attitudes (e.g., vaccine hesitancy) and behaviors (e.g., vaccine refusal) (12, 92). The prevalence of health mis-/disinformation has led scholars and health authorities to advocate for various intervention strategies to reduce its spread.

The science of public health communication, however, reveals many challenges to these efforts. Working definitions of health misinformation, such as “any health-related claim of fact that is false based on current scientific consensus” (26), highlight the challenge that scientific consensus can be elusive due to the partial and dynamic nature of the scientific enterprise. Take, for instance, changes in perceptions of the cost–benefit ratio of some cancer screening tests (65); changes in understanding whether COVID-19 spreads through droplets or aerosols (97); and evolving science on the value of cloth versus N95 masks for spreading airborne disease (79). Decades of communication and behavioral theory and research also make clear that not all information (whether true or false) is consequential in shaping intentions or behaviors (36). Consequently, efforts to both monitor and correct misinformation in the public communication environment can be costly, technically challenging at scale, and inefficient, particularly if the misinformation achieves limited exposure or has limited impact on relevant health behaviors (122). Finally, efforts to correct widespread or impactful misinformation are not always successful in the longer term, and the potential for these efforts to backfire or have unintended effects continues to be investigated (84, 125).

A robust body of research thus far has yielded some best-practice recommendations. First, misinformation spreads more quickly in a vacuum of clear and accurate information about a topic. While it may take some time for scientific knowledge about a novel health threat to emerge, public health communicators can fill this vacuum by communicating regularly with what is known (and with what level of certainty) and what is not currently known, along with what is being done to fill the knowledge gap (136). Second, in cases where a particular strand of misinformation is circulating widely and has plausible evidence of negative impact, clear and factual corrections that employ evidence-based methods can reduce false beliefs and the tendency to share them (94, 132, 135). Emerging evidence suggests that the impact of such corrections may hinge on the level of detail, repetition, and use of a trustworthy correction source (which varies by audience) (94). Third, there is strong potential for inoculation and prebunking strategies that seek to empower audiences to avoid misinformation, reject it upon exposure, and reduce the likelihood of sharing it (30, 103). Such strategies may have limited impact when audiences have already been heavily exposed to oppositional information, however (44); as such, these strategies may be best targeted to youth audiences to build broad media and information literacy skills (56). Fourth, there is a critical need to consider a variety of policy approaches that address the potential for actors to purposefully spread harmful disinformation about health topics for political or economic gain, with a particular need to curtail superspreaders of health misinformation (73, 128).

While these best practices are useful for effective public health communication planning, it is also vital to avoid laying the onus of change solely on information consumers. Just as nutrition is a joint function of both what consumers consume and what producers produce, a healthy communication ecosystem requires holding producers, conveyors, and platforms of information and communication responsible for their information evaluation policies.

## 3. PRACTICAL GUIDANCE FOR PUBLIC HEALTH COMMUNICATORS

Before moving to strategic decisions about the design, audience, or channel for messages or campaigns, communicators must first determine if communication, as one of many options for social action, is a reasonable primary solution for influencing a particular public health problem or behavioral outcome among a particular audience (43, 51). Alternative strategies might focus on enforcement, incentives, or compliance (107). While these alternative actions might benefit from communication efforts—for example, informing people about the existence of a prescription disposal program (147)—in such a case communication complements another implementation or dissemination strategy. Ineffective public health communication can result from efforts to influence outcomes for which communication is not an ideal primary solution. Not all problems, in other words, have communication solutions.

### 3.1. Practical Guidance for Message Development

Once investigators have established that public health communication is a useful strategy, message development and audience segmentation are key components of effective public health communication. Communication practitioners and researchers commonly employ theory to develop effective messaging that aims to change people’s intention to engage in a health behavior. Theories of behavior change and message effects can guide decisions about communication campaigns, inform decisions about strategic messaging, and inform effective segmentation of audiences by their beliefs and behaviors (37). Behavior change theories emphasize a variety of domains of beliefs (e.g., those pertaining to behaviors, risks, norms, and efficacy) that may be targeted by strategic messaging (36, 100). Communication scholars have developed an approach to systematically examine existing audience beliefs and use information about those beliefs to develop messages that are more likely to translate to behavioral changes (52). This approach suggests that researchers should use behavior change theory to inform the design of surveys to gauge (*a*) the proportion of people who do not endorse a particular belief at baseline, (*b*) the strength of the association between the potential targeted belief and the desired outcome, and (*c*) the extent to which the belief can reasonably be affected by a communication campaign (52). Other scholars emphasize the value of using surveys to develop a large set of candidate messages and use rapidly responsive data collection platforms to gauge perceived message effectiveness, which in turn can help to identify a subset of messages that are more likely to resonate with audiences (20, 82). While the predictive validity of these approaches remains a source of scholarly debate (85), both strategies offer communicators strategies to select promising content for messages disseminated at scale.

While behavior change theories can provide guidance on what to say, they offer little direction on how to say it. Message effect frameworks offer such guidance (37). Communication scholars have explored a variety of design considerations, such as the conditions under which personal stories outperform other forms of evidence (90), information on how to engage with opposing arguments (5), or guidance on whether to use threatening or humorous appeals (138, 140). While none of these strategies guarantees success, theory-informed strategies increase the likelihood of shifting beliefs, intentions, and/or behavior (86). These perspectives suggest that formative research informed by theory can improve strategic messaging choices to promote health behavior and emphasize the science involved in developing effective health communication.

### 3.2. Practical Guidance for Audience Segmentation

Effective public health communication also depends on acknowledging, interrogating, and understanding which audience factors could influence the success of a communication effort. Audience segmentation involves partitioning the target audience into smaller groups within a larger population according to meaningful distinctions, which may include their demographic or psychographic characteristics (118). Audience segmentation has long been recognized as essential to effective public health communication (115). Segmentation allows public health communicators to consider how the intersections of audience factors are likely to affect the delivery and outcomes of their communication efforts. Effective health communication requires acknowledgment of and strategizing about how to account for various audience factors across individual (e.g., media preferences, identity/similarity), interpersonal (e.g., social support, social capital), community (e.g., geographic disparateness, available resources, social norms), and policy (e.g., flexibility of systems to change, access to resources) levels.

Targeting refers to creating communications that consider specific population subgroups based on shared beliefs, characteristics, or identities (62, 109). Approaches to targeting strategy vary from simple identification of audiences by some defining characteristic deemed important for the communication efforts (e.g., demographics, psychosocial factors, media use) (61, 102) to more complex, data-driven techniques (e.g., latent class analysis) (119). Tailoring refers to creating communications that are individualized for members of an intended audience. Individualized messages for a population, community, or individual can increase the effectiveness of a communication campaign and stimulate positive behavior change (101). Tailoring employs strategies of personalization, feedback, and content matching (e.g., providing content based on recipient needs, deficits, or preferences), with the goal of enhancing message processing and impact (47).

Both tailoring and targeting could, for example, ensure that messaging aligns with the health literacy of a priority population. Health literacy is the degree to which people can find, understand, and use information to advise their health-related decisions (130). As indicated in Healthy People 2030, there are notable efforts to increase health literacy for individuals and populations (48). People have unprecedented access to health information, yet 9 out of 10 adults in the United States struggle with health literacy (78). Those with low health literacy are more likely to experience poor health outcomes, are less likely to receive preventive medicine, and struggle to manage chronic diseases. Targeting or tailoring messages for health literacy can facilitate the development of appropriate messages and visuals and determine which information sources should be used to provide messaging about public health.

To target or tailor a message, communicators often involve the intended audience or priority population in campaign development. This inclusion can be at any/all stages of a campaign, including efforts to understand the audience (e.g., primary/secondary research), message development, dissemination, and evaluation. This approach necessitates working with, rather than on, a priority population. Inclusion in communication development requires an exchange of ideas, knowledge, and experiences and includes mutual respect and reciprocity (54). However, approaches to inclusion and degrees of participation may differ necessarily depending on the situation and the needs of an intended population. For example, a community may have different needs or priorities that facilitate or impede their ability to work on a communication campaign. Priority shifts during the stages of campaign development are common (e.g., during the COVID-19 pandemic, historically underserved communities shifted focus from chronic disease prevention messaging to pandemic and vaccine messaging) (13). Community-based participatory research practices center community priorities, needs, and communication approaches. These community-centered approaches can help address health and power inequities. Communicators can also access important local knowledge, better understand the perceptions of a population, and assess behaviors that contribute to hazard exposure, often increasing the effectiveness of health messaging (14).

### 3.3. Guidance for Integrating Communication into Multilevel Public Health Interventions

Public health can further improve the effectiveness of efforts by integrating communication into multilevel health interventions. Continued and growing interest in using public health communication to address health disparities necessitates rethinking how communication strategies can be implemented effectively into existing interventions. For example, the National Institute on Minority Health and Health Disparities research framework explicates how levels of influence (e.g., individual and community) intersect with domains of influence (e.g., behavioral, sociocultural, health systems) (77). Relatedly, communication researchers and theorists have proposed different ways in which effective public health communication might be integrated into culture-centered, ecological, and multilevel approaches (31, 75, 88). For example, public health communication campaigns are often more successful if people exposed to campaign messages talk about those messages with other people (123). Conversation increases individual message exposure and reach and often, in turn, also positively affects the intended health behavior outcomes of interest (123).

The shift to considering the role of health communication across levels acknowledges that communication is itself a multilevel phenomenon and occurs within complex systems and information environments (59, 66). Recommendations for effective multilevel health communication can lean on theories of complex communication systems [e.g., communication infrastructure theory (143), the culture-centered approach (31), the theory of normative social behavior (99)] and plan projects that examine outcomes across levels of influence. Effective implementation of multilevel health communication interventions will require innovations in measures, methods, and training, as well as collaborations with implementation/dissemination scientists (88). Such efforts will inform effective health communication efforts to reduce health disparities and advance health equity.

## 4. EVALUATING COMMUNICATION INTERVENTIONS

As with most public health interventions, evaluation is a key component of public health communication programs. Indeed, principles that underlie the evaluation of public health communication campaigns are no different from those assessing other types of public health interventions. Several excellent texts speak to sound evaluation principles (51, 111), and hence we do not repeat them here. There are, however, some important considerations specific to public health communication interventions, the key points of which we discuss below.

A distinct component of public health communication evaluation pertains to the viability of running randomized controlled trials for attributing outcomes to the intervention in a causal manner. Not all health interventions use mass or social media; however, when they do, randomizing people to treatment and comparison groups presents a significant challenge and, in many instances, is an impossibility. If health communication messages are disseminated through broadcast television, for example, it may be possible to randomize different market segments into treatment and comparison groups, but this approach often requires a great deal of resources that many institutions, including regional public health departments, may be unable to marshal. Furthermore, given the osmotic nature of information across media—in that information appearing in one medium (e.g., broadcast television) can readily appear in another (e.g., social media or streaming platforms)—there is little guarantee that interventions delivered to one market serving as the treatment site will not diffuse to another site. The inability to randomize interventions and the likelihood of contamination among comparison sites have led evaluators of public health communication intervention to resort to statistical means for creating equivalence between treatment and comparison arms, without randomization. Propensity score matching, sensitivity analysis, and probit regression models are some of the methods that can be used for this purpose (4). Similarly, interrupted time series and regression discontinuity designs are yet other methods used to infer causality (19).

Public health communication scholars must also link exposure to the intervention with observed outcomes to make attributions to the intervention (51). Exposure is a necessary (though not sufficient) requirement for intervention outcomes, and lack of (repeated) exposure is among the most common reasons for communication campaign failures to promote social change (51, 137). Assessing exposure also allows researchers to determine if there is a dose–response relationship, an association between the magnitude of the exposure and that of the intervention outcomes, though measuring communication exposure reliably and validly is an enduring challenge (80, 116).

Evaluation, of course, is not limited to documenting health impacts. Health communication interventions also undergo rigorous formative assessments, the goal of which is to understand the facilitators of and barriers to change that may exist at the social, economic, political, and institutional levels. Similarly, process evaluations are undertaken to assess how the intervention is being rolled out, how it is being received, by whom, etc. The PRECEDE-PROCEED model is an oft-used framework that informs how formative assessments are undertaken and how interventions are deployed (41).

## 5. FUTURE DIRECTIONS

The interdisciplinary field of public health communication has much to offer public health research and practice. In closing, we identify areas for consideration, research, and investment to enhance the impact of strategic communication in a complex social, political, and cultural environment.

First, we note that many fields regularly contribute relevant knowledge to health communication theory and practice, and health communication scholars integrate multidisciplinary knowledge into their work. This relationship has not always been reciprocal, however, and we see little benefit to parochial boundaries to knowledge production or integration. As the emerging fields of dissemination science, data science, informatics, and misinformation studies gain momentum, we encourage scholars working in those spaces to consider the wealth of existing knowledge about communication processes and effects and avoid narrow and myopic efforts to reinvent the wheel.

Second, we share recent calls for behavioral scientists to integrate multilevel thinking into the design and evaluation of public health communication efforts. Doing so will necessitate a broader focus on community context, social diffusion, and institutional/public policy in these efforts. Failure to do so runs the risk of reinforcing dominant narratives of individual responsibility and blame for health inequalities and continuing to widen these disparities.

Third, such efforts should center on perspectives, lived experience, and situational knowledge from people and populations that have been historically excluded and minoritized. Like many fields, health communication has overgeneralized from Western, educated, industrial, rich, and democratic (WEIRD) populations and contexts. Failure to support, catalyze, and amplify knowledge from broader cultural and global contexts will limit the impact and relevance of public health communication in the future.

Fourth, the field must continue to evolve to understand and intervene in the dynamic, rapidly diffuse, networked, and increasingly user-generated media, information, and communication ecosystems of our time. Public health communication researchers will need to evolve to account for and integrate these changes into theory and practice to stay relevant and maximize public health impact.

Fifth, since public health communication is a process that rises beyond a communication product such as a brochure or a single message, it is also important to train members of the public health workforce on effective communication methods and techniques. This effort may involve, among the other core concepts described here, employing effective strategies to promote active listening, empathy, and understanding among health communicators within (e.g., doctors) and beyond (e.g., public health advocates) health care systems.

Finally, the field needs to broaden the investment and diversity of funding sources for public health communication research and intervention. COVID-19 revealed that billions of dollars of investment in scientific innovation alone do not come close to ensuring that people will adopt these innovations. To put it bluntly, scientists can develop the most effective vaccines and medical innovations known to humankind, but the impact of these innovations will be minimal if people do not accept them because they do not trust the scientists, doctors, public health practitioners, and elected officials who promote them. In our experience, communication strategy is brought into the conversation far too late in the process to maximize its impact, and far too few resources are spent on public health communication science in the first place. Recent efforts to increase such funding have also stalled due to politicization (108), an unfortunate outcome considering broadly recognized interest and the need for greater investment. We hope that this review conveys the breadth of existing public health communication knowledge that could be leveraged to increase the impact of public health in the future.

## SUMMARY POINTS

1. Research evidence, theory, and practical guidance produced from within and beyond the interdisciplinary field of health communication are well-positioned to help public health researchers, authorities, and advocates navigate the complex societal challenges that influence health and well-being in global contexts.
2. Reaching appropriate audiences with strategic messages from sources they consider credible, through channels they both use and trust, is neither formulaic nor static. Effectively reaching audiences requires inquiry, trial and error, and adaptations through learnings from research and evaluation.
3. The majority of health communication research has focused on the extent to which efforts influence individual-level outcomes, with far less scholarship focused on institutional or policy change as a vehicle for advancing public health. There is a need for greater theoretical development and research that center policy makers, policy advocacy, community mobilization, and power-building as pathways to policy change.
4. Health communication has the potential to reduce health inequities and advance social justice. However, it also risks widening disparities if sources of inequality are not considered, such as access and exposure to information and systemic barriers to behavior change.
5. Communication scholars have developed several approaches to inform message development and design, including survey-based methods to identify promising belief targets for intervention and randomized experiments using rapidly responsive data collection platforms to identify messages that resonate with audiences.
6. To develop communication strategies for specific audience segments, communicators often involve the intended audience or priority population in campaign development. This inclusion can be at any/all stages of a campaign, including efforts to understand the audience (e.g., primary/secondary research), message development, dissemination, and evaluation. This approach necessitates working with, rather than on, a priority population.
7. Public health can further improve the effectiveness of efforts by integrating communication into multilevel health interventions. Communication is itself a multilevel phenomenon and occurs within complex systems and information environments.
8. Practical and conceptual challenges to conducting randomized interventions necessitate that evaluations of public health communication interventions often resort to statistical means for creating equivalence between treatment and comparison groups. Measuring exposure to communication interventions allows researchers to determine if there is an association between the magnitude of the exposure and intervention outcomes, though measuring communication exposure reliably and validly is an enduring challenge.

## FUTURE ISSUES

1. Many fields regularly contribute relevant knowledge to public health communication theory and practice, and health communication scholars have integrated multidisciplinary knowledge into their work. This relationship has not always been reciprocal. Scholars in disciplinary and emerging multidisciplinary fields who are working on public health communication topics should consider the breadth and wealth of existing and continually emerging knowledge about communication processes and effects.
2. Integrating multilevel thinking into the design and evaluation of public health communication efforts has great potential benefits. This work will necessitate a broader focus on community context, social diffusion, and institutional/public policy in these efforts. Failure to do so risks reinforcing dominant narratives of individual responsibility and blame for health inequalities and continuing to widen these disparities.
3. Public health communication has overgeneralized from WEIRD populations and contexts. Failure to support, catalyze, and amplify knowledge from broader cultural and global contexts will limit the impact and relevance of public health communication in the future.
4. The field should continue to evolve to understand and intervene in the dynamic, rapidly diffuse, networked, and increasingly user-generated media, information, and communication ecosystems of our time. Research must evolve to integrate these changes into theory and practice to stay relevant and maximize public health impact.
5. Broadening investment in and the diversity of funding sources for public health communication research and intervention is an urgent and critical need. There is a far higher likelihood of success when communication strategy is brought into the conversation at the start rather than at the end of the process of developing public health policies and interventions.

## Abbreviations

Misinformation (about health): any health-related claim of fact that is false based on current scientific consensus (definition from Chou et al.)
Disinformation (about health): health-related claim of fact that is false based on current scientific consensus and that is disseminated with the intent to misinform or deceive (definition from Chou et al.)
Audience: entity for whom the information is intended, which could be individuals or groups/organizations
Audience segmentation: partitioning the potential audience of communication efforts into smaller groups by meaningful distinctions (demographic, psychographic, etc.) (see research from Smith)
Channel (of communication): the mechanism, medium, or platform through which information is exchanged, which could be one-on-one, one-to-many, or many-to-many
Process (communication as): the meaning and impact of communication vary over time and according to characteristics of the audience, channel, source, and social context
Source (of communication): the people, organizations, or platforms who produce, transmit, or retransmit information and messages
Effectiveness (of communication): occurs when planned communication activities achieve a priori stated expectations or objectives related to outcomes of interest
Effects (of communication): occur when a communication activity results in a detectable difference in an audience on one or more outcomes of interest
Outcome (communication as): the product of a communication process (e.g., a pamphlet) or communication behaviors resulting from strategic communication (e.g., increased conversation)
Structural interventions: efforts to improve health outcomes by affecting policies, systems, or structures that shape opportunities for health and well-being
Health equity: a state in which everyone has a fair and just opportunity to attain their highest level of health (definition from the US Centers for Disease Control and Prevention)
Exposure: the extent to which audience members saw, heard, or otherwise interacted with a communication effort, which may be direct or indirect
Knowledge gap: the idea that those with greater resources acquire knowledge from communication exposure at a higher rate than those with fewer resources
Targeting: creating or producing information and messages that consider specific population subgroups based on shared beliefs, characteristics, or identities (definition from Kreuter & Wray, Schmid et al.)
Tailoring: creating or producing information and messages that are individualized for members of an intended audience (definition from Rimer & Kreuter)
Multilevel health communication: the idea that health communication occurs across individual, interpersonal, cultural, and societal levels and can have influence within and across those levels
